# Treatment strategies in eating disorders with comorbid conditions and in under-represented clinical populations

**DOI:** 10.1192/j.eurpsy.2024.98

**Published:** 2024-08-27

**Authors:** G. Paslakis

**Affiliations:** University Clinic for Psychosomatic Medicine and Psychotherapy, Ruhr-University Bochum, Campus East-Westphalia, Luebbecke, Germany

## Abstract

**Abstract:**

Eating disorders (EDs) have long been thought to be conditions that only or mainly affect women, especially young, affluent, skinny girls and women in Western cultures. Mostly over the last decade, we have come to realize that EDs may affect individuals of all genders, ages, sexual orientations, ethnic, and socio-economic backgrounds. This, in turn, has implications for ED presentation and assessment, and the necessity for adjustments in the provided care according to diverse treatment needs. Here, we present and discuss current advances in ED-related research in underrepresented groups as well as the need to further incorporate diversity aspects in clinical care and research within the ED realm.

**Disclosure of Interest:**

None Declared

